# A Phase II, Open-Label Study of Lenalidomide and Dexamethasone Followed by Donor Lymphocyte Infusions in Relapsed Multiple Myeloma Following Upfront Allogeneic Stem Cell Transplant

**DOI:** 10.3390/curroncol31110535

**Published:** 2024-11-16

**Authors:** Richard LeBlanc, Stéphanie Thiant, Rafik Terra, Imran Ahmad, Jean-Sébastien Claveau, Nadia Bambace, Léa Bernard, Sandra Cohen, Jean-Sébastien Delisle, Silvy Lachance, Thomas Kiss, Denis-Claude Roy, Guy Sauvageau, Jean Roy

**Affiliations:** 1 Division of Hematology, Oncology and Transplantation, Department of Medicine, Maisonneuve-Rosemont Hospital, Montréal, QC H1T 2M4, Canada; richard.leblanc.1@umontreal.ca (R.L.); rterra.hmr@ssss.gouv.qc.ca (R.T.); imran.ahmad.1@umontreal.ca (I.A.); js.delisle@umontreal.ca (J.-S.D.); silvy.lachance@umontreal.ca (S.L.); thomas.kiss@umontreal.ca (T.K.); denis-claude.roy@umontreal.ca (D.-C.R.); guy.sauvageau@umontreal.ca (G.S.); 2 Centre de Recherche, Hôpital Maisonneuve-Rosemont, Montréal, QC H1T 2M4, Canada; 3 Faculty of Medicine, Université de Montréal, Montréal, QC H2V 0B3, Canada

**Keywords:** allogeneic hematopoietic cell transplant, donor lymphocyte infusion, minimal measurable disease, multiple myeloma

## Abstract

Background: To date, the only potential curative treatment for multiple myeloma (MM) remains allogeneic (allo) hematopoietic cell transplant (HCT), although, most patients will eventually relapse. In relapsed patients, donor lymphocyte infusions (DLIs) have been reported to control disease, but the optimal strategy prior to and doses of DLIs remain unclear. With this study (NCT03413800), we aimed to investigate the efficacy and toxicity of lenalidomide and dexamethasome (Len/Dex) followed by escalating pre-determined doses of DLIs in MM patients who relapsed after allo HCT. Methods: Patients aged 18–65 years with relapsed MM following upfront tandem autologous (auto)/allo HCT were eligible. Treatment consisted of six cycles of Len/Dex followed by three standardized doses of DLIs: 5 × 10^6^ CD3+/kg, 1 × 10^7^/kg and 5 × 10^7^/kg every 6 weeks. Bone marrow minimal measurable disease (MRD) using flow cytometry (10^−5^) was performed at enrolment, then every 3 months for 2 years or until disease progression, in a subset of patients. The primary endpoint was efficacy as measured by progression-free survival (PFS) at 2 years following Len/Dex/DLIs. Secondary objectives were safety including GVHD, response including MRD status and overall survival (OS). Results: A total of 22 patients participated in this study, including 62% with high-risk cytogenetics. With a median follow-up of 5.3 years (range: 4.1–6.1), PFS and OS were 26.5% (95% CI: 10.4–45.9%) and 69.2% (95% CI: 43.3–85.1%), respectively. Overall, the best responses achieved post-Len/Dex + DLIs were complete remission in 9.1%, very good partial response in 50%, and progressive disease in 40.9%. Among the nine patients tested for MRD, only two achieved a negative status after receiving DLIs. Six patients died, all due to disease progression. No acute GVHD was observed after DLIs. We report a very low incidence of moderate/severe chronic GVHD of 18.2% with no need for systemic immunosuppressants one year after diagnosis. No unexpected adverse events were observed. Interestingly, a positive correlation between response to Len/Dex re-induction and response to DLIs was found (*p* = 0.0032). Conclusions: Our findings suggest that Len/Dex/DLIs in second line treatment after upfront tandem auto/allo HCT in relapsed MM patients remains feasible and safe. With a potential correlation between induction chemotherapy and DLI responses, more potent induction regimens together with higher doses of DLIs should be considered in the future.

## 1. Introduction

Multiple myeloma (MM) remains a morbid disease associated with a poor outcome, particularly for patients with adverse biological factors. To date, allogeneic (allo) hematopoietic cell transplant (HCT) remains the only potentially curative option for MM patients, with reported long-term survival ranging between 20 and 40% in the literature [1,2,3]. In the first-line setting, approximately 50% of newly diagnosed MM patients who undergo tandem autologous (auto)/allo HCT will eventually relapse within the first 5 years of transplant [1,3]. Nevertheless, several clinical observations support the existence of a graft-versus-myeloma (GvM) effect [4,5], including achievement of prolonged remission in allografted patients with advanced disease [2,6,7,8,9,10,11,12,13].

One possible therapeutic modality in relapsed MM after allo HCT is donor lymphocyte infusion (DLI). DLIs alone have been shown to induce response rates in 36–61% of patients with MM [4,14,15,16,17,18,19,20,21,22]. In the first publications, T-cell dose > 1 × 10^8^ cells/kg and occurrence of graft-versus-host disease (GVHD) were associated with a better response [18,19,22]. The GvM effect has indeed been associated with chronic GVHD and a reduction in incidence of relapse [23]. The ability of donor lymphocytes to produce an allogeneic immune reaction is also associated with antibody responses against highly expressed myeloma-associated antigens [24].

Given the remarkable activity of immunomodulatory drugs (IMiDs) and proteasome inhibitors, as well as reports suggesting that immunotherapy is mostly effective in the setting of minimal measurable disease (MRD), few studies have examined the effect of reinduction therapy in association with DLIs. The goal is to maximally decrease tumor burden with reinduction chemotherapy before DLIs to induce a synergistic effect and improve response. Induction therapies before DLIs have however been widely variable from one protocol to another, ranging from a combination of vincristine, adriamycine, and dexamethasone (VAD) [19,22]; lenalidomide (Len) [25,26]; thalidomide [25,27]; and dexamethasone (Dex) in monotherapy [22,25], in combination with interferons [25], in bortezomib monotherapy, [25] or associated with Dex [28]. In previous publications, DLIs were also highly variable in terms of doses (ranging from 1 × 10^6^ to 5 × 10^8^ T-cells/kg), number of infusions (from 1 to 4), and timing after transplant.

IMiDs are of particular interest in counteracting the immunosuppression observed in MM, as they have been reported to stimulate activities of CD4+, CD8+, natural killer, and dendritic cells both in vitro and in vivo [29,30,31,32,33]. Len is indeed a potent stimulator of T-cell proliferation following primary induction by T-cell receptors through increased production of IL-2, IL-10, and IFN-gamma. Moreover, it exhibits dose-dependent inhibition of IL-1 and IL-6 production, as well as modulation of IL-12 levels [31,34,35,36,37,38].

One important concern with the use of Len after allo HCT is the occurrence of GVHD [39], although more recent data point toward an anti-GVHD effect [38]. The efficacy of Len and Dex combination (Len/Dex) in relapsed MM outside the allo HCT setting has been demonstrated in two randomized, double-blind, placebo-controlled trials, MM-009 and MM-010 [40,41,42]. Based on these results, we hypothesized that the cytoreductive and immunomodulatory effects of Len would induce a permissive immunological environment, promoting the immunotherapeutic activity of DLIs, while the combination with Dex would synergize the efficacy of Len and reduce the risk of GVHD. We therefore designed a two-step strategy of increasing doses of Len in association with Dex, followed by escalation of DLI doses to offer optimal disease control in relapsed MM patients after tandem auto/allo HCT.

## 2. Materials and Methods

### 2.1. Study Design and Participants

This prospective phase II study was conducted at Hôpital Maisonneuve-Rosemont (HMR), affiliated to Université de Montréal, Québec, Canada. This research was performed in accordance with the Declaration of Helsinki. Informed consent was obtained from all patients, and this study was approved by our institutional review board (ClinicalTrial.gov: NCT03413800).

Patients 18–65 years old with relapsed MM following upfront tandem auto/allo HCT were eligible. They all received G-CSF-mobilized peripheral blood stem cells from 8/8 matched-sibling or unrelated donors. Initially, measurable disease based on the IMWG criteria at time of relapse was mandatory to participate. The protocol was subsequently amended to include patients with oligo/non-secretory disease if ≥ one positive myeloma lesion on positron emission tomography (PET) scan was documented. Patients who met the eligibility criteria but refused to participate in this study because of serial bone marrow (BM) examinations were offered to be treated similarly with the Len/Dex induction regimen followed by increasing doses of DLIs. Cytogenetic was considered at high risk when at least one of the following abnormality was positive in ≥10% purified plasmocytes by FISH: del17p, t(4;14), t(14;16); t(14;20); and abnormality of chromosome 1. Patients who had been previously exposed to Len after allo HCT, refractory to Len at any time before HCT or known with hypersensitivity to Len, were excluded. Other exclusion criteria were ≥ active grade II acute GVHD, severe chronic GVHD, a Karnofsky score < 70%, and poor organ function.

### 2.2. Induction with Lenalidomide, Dexamethasone, and DLIs

Patients were scheduled to receive a total of six cycles of Len/Dex followed by three DLIs. Len was administered for 21 consecutive days of each 28-day cycle. The initial Len dose was 10 mg orally, with a 5 mg increment after each cycle to a maximum dose of 25 mg daily to minimize the potential risk of acute GVHD. Dex was administered orally at a dose of 40 mg weekly, except in underweight patients (body mass index < 18.5), who received 20 mg weekly. Dose adjustments were allowed based on renal function for Len [43] and toxicity attributed to Len or Dex. During Len/Dex treatment, patients who developed a ≥ grade II acute GVHD, severe chronic GVHD, or progression at any given time had both Len and Dex discontinued and became ineligible to initiate or receive additional DLIs. Four to six weeks after the end of the last cycle of Len, eligible patients started DLIs at 5 × 10^6^, 1 × 10^7^, and 5 × 10^7^ CD3+ cells/kg for a maximum of three doses spaced 6 weeks apart. Len maintenance was not restarted after DLIs.

### 2.3. Patients’ Follow-Up

**Figure 1:**
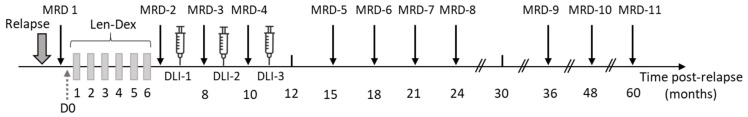
Overall schedule of treatments and measurable residual disease (MRD) assessments.

During the follow-up phase, starting after the last DLI, clinical evaluations were performed every 4 weeks for 3 months, then every 3 months up to 5 years after initiation of the first Len/Dex cycle. At each visit, patients were evaluated with a complete physical examination and complete blood counts with differential and serum biochemistry. Disease status was monitored with serum protein electrophoresis and immunofixation, serum-free light chains, and a 24 h urine collection for Bence Jones at each visit. Chimerism studies were performed yearly. A BM aspirate was performed at baseline, +6, +8, and +10 months post-Len/Dex before each DLI infusion; every 3 months during the first-year post DLI-3; then at +24, +30, +36, +48, and +60 months; and at relapse to evaluate MRD status (Figure 1). Patients with oligo/non-secretory disease with documented plasmacytomas at relapse had a PET scan performed every 3 months for one year, then every 6 months for one year and at time of suspected progression.

Acute GVHD was evaluated using modified Glucksberg [44] and IBMTR criteria [45], whereas chronic GVHD was evaluated using NIH criteria [46] at each visit. MM responses were categorized according to the International Myeloma Working Group (IMWG) criteria [47]. For patients with oligo/non-secretory disease, response categorization also included Lugano classification [48]. All adverse events (AEs) were assessed using NCI CTCAE v.4.0. Patients not on protocol NCT03413800 were followed using the same criteria except for serial BM MRD evaluations.

### 2.4. MRD Assessment

MRD assessment by Next-Generation Flow cytometry (NGF) was performed according to the EuroFlow consortium standards in the HMR flow cytometry laboratory as previously described [49,50]. Patients were considered as MRD negative if phenotypically abnormal plasma cells were <30 cells in at least one BM sample.

### 2.5. Endpoints and Statistical Analysis

The primary objective of this phase II clinical study was to determine the efficacy of six cycles of Len/Dex followed by three DLIs at increasing doses in patients with relapsed myeloma after allo HCT, as measured by progression-free survival (PFS) 2 years after the last DLI. Secondary objectives included incidences of grade ≥ III non-hematologic toxicity and grade ≥ IV hematologic toxicity, incidences of acute GVHD at 6 months and one year after last DLI, and chronic GVHD at 6 months, one year, and two years after last DLI. Secondary objectives also included response rates based on IMWG criteria, including MRD evaluation; NRM at 6, 12, and 24 months; overall survival (OS); and PFS. OS and PFS were estimated using the Kaplan–Meier method. Statistical analyses were carried out using R software (v4). Datalock was performed on 27 March 2024.

## 3. Results

### 3.1. Patients and Transplant History

Between July 2017 and June 2022, twenty-three patients consented to this prospective phase II study. Ten patients accepted the successive BM exams for MRD measurements, while thirteen patients received the same treatment procedure without undergoing repetitive BM examinations. Among these twenty-three patients, one patient unable to receive DLIs due to donor unavailability was excluded from the analysis. Patients’ and disease characteristics at time of diagnosis, in addition to details on first line treatment, are described in Table 1. Median age at allo HCT was 49 years (range: 30–62), 54% were males, and most patients (73%) had a low HCT comorbidity index of 0–1 (Table 2). An International Staging System score of III was found in 23%, and 62% of patients tested had high-risk cytogenetics (10/16 evaluated patients). At the time of the first line treatment, most (82%) patients received an induction treatment with CyBorD, then standard of care in Canada, and reached either a partial response (PR) (41%) or a very good partial response (VGPR) (59%). Half of the patients were transplanted with an 8/8 HLA-matched sibling donor following a nonmyeloablative regimen consisting of fludarabine 90 mg/m^2^ and cyclophosphamide 1500 mg/m^2^, while the other half received fludarabine 90 mg/m^2^ and 2 Grays of total body irradiation with an infusion of an 8/8 HLA-matched unrelated donor graft. A median of 8.1 × 10^6^ CD34+ cells/kg was infused. Only three out of twenty-two patients developed acute GVHD after allo HCT: two patients with grade II (D + 43, D + 101) and one with late grade III (D + 117). Ten patients experienced mild (*n* = 5) or moderate (*n* = 5) chronic GVHD. Almost all (21/22) patients reached ≥ VGPR after allo HCT. Median time of relapse post allo HCT was 22.6 months (range: 2.7–120.6), at a median age of 51 years (range: 32–64).

### 3.2. Len/Dex Treatments and DLI Infusions

At time of relapse, all but one patient presented with 100% chimerism; one patient relapsed on day + 80 (chimerism 77%) (Table 3). In total, nineteen patients received six cycles of Len/Dex as per protocol, with a median time between allo HCT and initiation of Len of 23.4 (range: 3.7–124) months. Deviations from the planned treatment schedule occurred in three subjects. One presented with grade III nausea and anorexia within the first 2 weeks of Len, which was replaced by thalidomide 200 mg daily. Another patient received twelve cycles of Len/Dex due to unexpected delays in donor lymphocytes collection. Finally, one patient received daratumumab in addition of Len/Dex. Except for patients presenting early relapses after Len/Dex (*n* = 4) or active GVHD (*n* = 1), all others received three escalated doses of DLIs 6 weeks apart at the dose of 5 × 10^6^, 1 × 10^7^ and 5 × 10^7^ CD3+ cells/kg. In 4 patients, the third dose was lower than planned due to limitations in the number of CD3+ cells provided by the collecting center. One patient received a slightly higher first dose of 8.9 × 10^6^ CD3+ cells/kg, while another received a slightly higher third dose at 7 × 10^7^ CD3+ cells/kg.

### 3.3. Safety

Grade ≥ III non-hematologic AEs reported from the time of the first cycle of Len/Dex until up to one year after the last DLI or time of progression are shown in Table 4. No grade IV non-hematologic AEs were observed, and no patient died due to an AE. Unsurprisingly, infections remained the most common complications but were easily controlled with appropriate treatment. In terms of second primary malignancies, two patients presented with basal cell carcinoma after DLI infusions.

### 3.4. Responses and Minimal Residual Disease

Among the nine patients (one excluded due to permanent donor unavailability to give DLIs) who had serial bone marrow examinations, two achieved CR. Globally, the best response achieved following induction with Len/Dex was 68.2% with ≥ VGPR, 27.3% with PR, and 4.5% with stable disease. Following DLIs, 59.1% of patients achieved ≥ VGPR, and 40.9% presented a progressive disease during or immediately after DLIs. There was no correlation between the best response to DLIs and best response achieved after allo HCT (Spearman R = 0.2159, *p* = 0.3346) or time to progression after allo HCT (Spearman R = −0.2540, *p* = 0.2665) (Table 1). However, we found a positive correlation between response to Len/Dex induction and response to DLIs (Spearman R = 0.5996, *p* = 0.0032). The MRD results for the nine patients with BM examination are shown in Table 5. Among them, five had negative MRD after receiving Len/Dex, before receiving a first DLI. In two patients, MRD remained positive after Len/Dex; one patient remained MRD+ after three DLIs, while the other had a substantial decrease in clonal plasma cells after two DLIs. Interestingly, this was the only patient who developed chronic GVHD one month after the last DLI. Finally, two patients relapsed before receiving their first infusion.

### 3.5. GVHD, Survival, and Relapse

GVHD characteristics are presented in Table 3. Only one patient developed grade I (skin) acute GVHD during the second cycle of Len; this patient had previously developed grade II cutaneous acute GVHD on D + 42 after allo HCT. No acute GVHD was observed after DLI infusions. Only five patients (23%) developed chronic GVHD, including two who had previously developed moderate chronic GVHD post allo HCT. They both remain in VGPR two years after beginning Len. We did not observe any correlation between the occurrence of GVHD after allo HCT and after DLIs.

**Figure 2:**
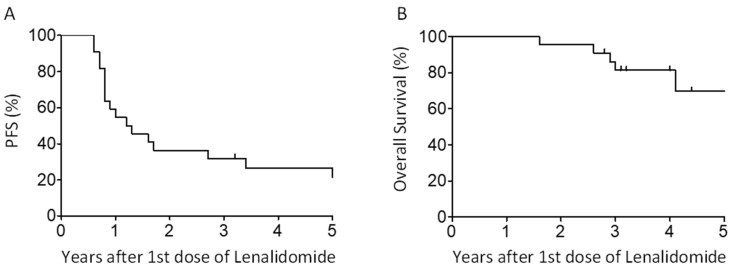
Kaplan–Meier estimates of (**A**) progression-free survival and (**B**) overall survival after initiation of first cycle of lenalidomide.

After a median follow-up of 5.3 (range: 4.1–6.1) years, most patients (77.3%) have experienced progression of their disease at a median of 1.2 years (range: 0.8–3.3) after initiation of the first Len cycle. Patients with high-risk cytogenetics had a median time of progression of 0.8 year (range: 0.6–1.1) versus 5.2 years (range: 0.9–7.7) for low-risk patients, which was significantly shorter (Wilcoxon Mann–Whitney test, *p* = 0.0027 **) (Table 3). At two years post 1st cycle of Len, PFS and OS were 36.4% (95%CI: 17.4–55.7%) and 95.5% (95%CI: 71.9–99.3%), respectively. At five years, PFS and OS were 26.5% (95%CI: 10.4–45.9%) and 69.2% (95%CI: 43.3–85.1%), respectively (Figure 2). Most patients who relapsed received a daratumumab-based salvage therapy (Table 6). At time of datalock, 45% of patients were not refractory to any proteasome inhibitor (PI), IMIDs, and anti-CD38 antibody after their first two lines of treatment and beyond. Six patients have died, all from refractory myeloma, and none from treatment with Len/Dex or DLIs (NRM 0% at 2 years).

## 4. Discussion

The best treatment strategy in MM patients relapsing after allo HCT remains unclear. There are currently no guidelines on agents to be used for optimal cytoreduction, timing, dose and schedule of DLIs. Compared to previously published studies on DLIs following allo HCT [18,19,22,26,28], ours used a homogeneous induction treatment followed by similar DLI escalation doses for all patients. Patients on Len/Dex achieved an overall response rate of 95.4% including 68.2% with ≥ VGPR. With this approach, after a median follow-up of 5.3 years, the median PFS was 1.2 years (range 0.8–3.3 years) and the median OS has not been reached. We observed that patients with high-risk cytogenetics progressed more rapidly after Len/Dex/DLIs.

The 2-year PFS and OS in our study are consistent with other reports using a combination of IMIDs or PIs followed by DLIs, although it is difficult to compare the results due to the great heterogeneity in therapies. El Cheikh et al. treated nine MM patients with progressive or residual disease at D + 100 post allo HCT with a combination of Len/DLIs. Patients received a median of six cycles (range: 1–10 cycles) of Len with dosage ranging from 10 to 25 mg followed by 1–3 escalating doses of DLIs every 3 months (#1: 1 × 10^7^, #2–3: 1 × 10^8^ T-cells/kg). The 2-year PFS and OS were 50% and 69%, respectively, similar to our study [26]. Montefusco et al. treated sixteen MM patients in relapse post allo HCT with a combination of bortezomib/Dex and 1–4 doses of DLIs. The 2-year PFS and OS were 37% and 79%, respectively [28]. Kröger et al. presented a higher 2-year estimated OS and PFS at 100% and 84%, respectively, in eighteen MM patients receiving low-dose thalidomide and DLIs. Several factors can impact response to DLIs such as tumor burden at time of infusion, cytogenetics, interval between transplant and DLIs, donor/recipient sex match and donor source (sibling vs. unrelated) as reported in other hematological malignancies [51,52] but not described in the three publications discussed above. Beitinjaneh et al. reported a poor response to DLIs (7%) in fifteen MM patients with progressive disease post allo HCT with extramedullary disease, possibly due to low access of T cells to the soft tissues, a frequent site of relapse after allo HCT [25]. In our cohort, four patients presented plasmacytomas at relapse (two patients in bone, one patient in soft tissues, and one in both bone and soft tissues). Post DLIs, one of them was in CR, two were in VGPR, and one had progressive disease. In Lokhorst et al.’s study, 13/27 patients received re-induction therapy before DLIs consisting of VAD, Dex, or intermediate dose melphalan. They showed that positive response to reinduction is a predictive factor for DLI response.

We observed a positive correlation between response to Len/Dex reinduction and DLI responses in our cohort. The efficacy of DLIs to treat relapse post allo HCT has been shown to be related to tumor load in myeloid malignancies [19,53,54,55]. Patients who cannot achieve at least VGPR or preferably better after reinduction should likely be treated with additional cycles or a combination of different agents to achieve a low tumor burden before receiving DLIs. Both Lokhorst et al. and Salama et al. have reported that more than 85% of responders have experienced acute or chronic GVHD [18,20,22] following DLIs, with several of their patients developing ≥ grade III acute GVHD or extensive chronic GVHD. Similarly, Van de Donk et al. treated sixty-three patients with 1–4 DLIs with doses ranging from 1 × 10^6^ to 3 × 10^8^ T cells/kg and showed a significant association between response and GVHD, with 66.7% of responders developing acute GVHD and 63% chronic GVHD. Among them, seven patients (11.1%) died, including five from GVHD^19^. Lokhorst et al. and Salama et al. also showed a correlation between T cell dose ≥ 1 × 10^8^ cells/kg and response to DLIs [18,22]. Due to our low occurrence of GVHD in our cohort, we cannot confirm this observation. Patients in our cohort also received a maximum dose of 6.5 × 10^7^ T cells/kg in total, well below the threshold of 1 × 10^8^ cells/kg previously used, which may also explain why we did not observe the same GVHD outcomes. The main difference between early studies and the most recent ones [26,27,28], including ours, lies in the use of DLI alone [18,19] or after reinduction with other agents. It has been suggested that GVHD could potentially be impacted by the use of IMiDs or PIs [56,57,58,59]. The high responses of relapsed MM patients to the combination of DLIs with thalidomide in Kröger et al.’s study, coupled with a low incidence of GVHD, suggests that the GvM effect and GVHD could be managed independently [27]. Further investigations need to be conducted in MM patients to identify specific factors influencing DLI response.

In modern second line therapy after auto HCT followed by Len maintenance, a retrospective analysis demonstrated that daratumumab-containing regimens achieved a median PFS of 15.8 months [60]. Specifically, median PFS was 21.7 months using DRd (daratumumab, Len, Dex) in second line, 12.9 months with DVd (daratumumab, bortezomib, Dex), and 18.9 months with DPd (daratumumab, pomalidomide, Dex) [60]. Contemporary therapies at relapse have resulted in prolonged median PFS using DRd (44.5 months) [61], DVd (16.7 months) [62], PVd (11.2 months) [63], DKd (28.6 months) [64], IsaKd (35.7 months) [65], DPd (12.4 months) [66], and IsaPd (11.5 months) [67]. Used in second line, these regimens induced greater clinical benefits. For example, in the OPTIMISMM study, median PFS was 17.8 months, while it was 11.2 months overall [68], similar to the observations of CANDOR and IKEMA studies [69,70]. Our results in terms of PFS are inferior to many of those obtained using more contemporary treatments. However, our study population was enriched with high-risk cytogenetic patients and received what could be considered today as a suboptimal induction regimen with Len/Dex. With a potential correlation between induction chemotherapy response with DLI response as we report, we could expect that using more effective drug combinations for induction therapy and achieving sustained MRD on BM examination prior to DLIs, along with higher doses of DLIs could optimize the impact of this therapeutic approach.

In our study, the Len and Dex doses were escalated to a maximum of 25 mg daily (21 days out of 28) and 40 mg weekly, respectively, to improve tolerance and to minimize the risk of acute GVHD. Only one patient developed systemic symptoms with Len and had to be switched to thalidomide. Len has been reported to induce high rates of acute GVHD after allo HCT, ranging from 38 to 60% when used as maintenance therapy [37,71,72]. In contrast, other investigators have observed an incidence ranging from none to 37%, similar to patients allotransplanted never exposed to Len [26,39,56]. In our cohort, only one patient presented a grade I cutaneous acute GVHD while receiving Len, which easily resolved with topical steroids. We hypothesize that the low rate of acute GVHD observed in our patients could be related to the late timing of Len initiation post allo HCT (median of 22.6 months) and the concomitant use of Dex inducing immunosuppression. After DLIs, 20 grade III non-hematologic adverse events were reported, most of which were fully reversible and without sequelae; there was no grade IV and no death related to DLI.

The BM microenvironment in MM is known to be highly immunosuppressive and can decrease anti-tumor immunity, thereby contributing to tumor progression [73]. Myeloma cells are known to evade the immune system by several mechanisms including reduced HLA expression, reduced tumor antigens, increased expression of T-cell inhibitory ligands such as programmed cell death ligand-1 (PD-L1) and PD-L2, recruitment of Tregs, myeloid-derived suppressor cells (MDSCs), and dendritic cells [74]. Dendritic cells accumulate in the BM where they protect tumor plasma cells from CD8+ T-cell killing [75,76]. Furthermore, Franssen, et al. have demonstrated that relapsed MM patients post allo HCT and unresponsive to DLIs had a significantly higher level of CD14+ MDSCs, CD14- MDSCs and Tregs. Higher frequencies of Tregs, but not of MDSCs, were significantly associated with DLI unresponsiveness and shorter PFS and OS [77]. These immunosuppressive mechanisms are thought to challenge the initiation and maintenance of the anti-MM immunotherapy sought after DLIs. In the future, pre-DLI Treg depletion might become a promising strategy for improvement of DLI outcomes in MM patients [73], as well as potential donor-derived CAR-T cells and combination with bispecific antibodies.

Our study has several limitations, including a small number of patients, some heterogeneity in induction therapy and DLI doses, and that only a subset of patients agreed to have serial BM exams.

## 5. Conclusions

We report that using Len/Dex followed by three escalated doses of DLIs as second line treatment in relapsed MM after tandem auto/allo HCT is safe with a low risk of GVHD. Responses in our patients, albeit similar to most previously published studies, were associated with a disappointingly shorter PFS compared to modern regimens. In the future, achieving better response, including MRD negativity after triplet induction, is likely to improve the clinical benefits of DLIs. Finally, better characterization of prognostic factors impacting response to DLIs could help to identify which patients might benefit most DLIs.

## Figures and Tables

**Table 1:** Myeloma characteristics and initial treatments.

	N = 22 (%)
Median age at diagnosis in years (range)	48 (30–61)
Male	12 (54)
Median creatinine at Dx in µmol/L (range)	84 (54–414)
Median % of plasma cells at Dx (range)	50 (5–90)
Isotype	
IgG kappa	6 (27)
IgG lambda	3 (14)
IgA kappa	4 (18)
IgA lambda	3 (14)
IgM kappa	1 (4)
FLC kappa	3 (14)
FLC lambda	2 (9)
International staging system at diagnosis	
I	8 (36)
II	9 (41)
III	5 (23)
Cytogenetics available	16 (73)
Among patients tested	
No high-risk abnormality	6 (38)
t(4;14)	2 (12)
del17p	1 (6)
Gain 1q	4 (25)
≥2 cytogenetic abnormalities	3 (19)
Induction prior to HCT	
CyBorD	18 (82)
Bor/Dex	2 (9)
Bor/Dex + Len/Dex	2 (9)
Median number of induction cycles (range)	4 (4–9)
Best response to 1st line of treatment	
VGPR	13 (59)
PR	9 (41)
Maintenance after HCT	
None	17 (77)
Lenalidomide	2 (9)
Bortezomib	1 (4)
Unknown	2 (9)

Bor: bortezomib; CyBorD: cyclophosphamide, bortezomib, dexamethasone; Dex: dexamethasone; Dx: diagnosis; FLC: free light chain; HCT: hematopoietic cell transplant; Len: lenalidomide; PR: partial response VGPR: very good partial response.

**Table 2:** Allogeneic transplant characteristics and outcome.

	N = 22 (%)
Median age at allogeneic HCT in years (range)	49 (30–62)
HCT-CI	
0–1	16 (73)
2–3	6 (27)
Donor	
Matched sibling	11 (50)
Matched unrelated donor	11 (50)
Recipient CMV status	
Negative	14 (64)
Positive	8 (36)
Donor CMV status	
Negative	17 (77)
Positive	5 (23)
Conditioning regimen	
FluCy	11 (50)
FluTBI 2 Gy	11 (50)
Allogeneic graft	
Median CD34+cell × 10^6^/kg (range)	8.1 (4.2–11.9)
Median TNC × 10^8^/kg (range)	8.9 (2.6–18.5)
Acute GVHD incidence at D + 180	
None	19 (86)
I–II	2 (9)
III–IV	1 (4)
Chronic GVHD (highest grade until relapse)	
None	12 (54)
Mild	5 (23)
Moderate	5 (23)
Severe	0
Best response after allogeneic HCT	
Progressive disease	1 (4)
VGPR	9 (41)
CR	3 (14)
sCR	9 (41)
Median time of relapse post allogeneic HCT in months (range)	22.3 (2.7–120.6)
Median age at relapse in years (range)	51 (32–64)

CI: comorbidity index; CMV: cytomegalovirus; CR: complete remission; Flu: fludarabine; GVHD: graft-versus-host disease; Gy: gray; HCT: hematopoietic cell transplant; sCR: stringent complete remission; TBI: total body irradiation; TNC: total nucleated cell; VGPR: very good partial response.

**Table 3:** Donor lymphocyte infusion characteristics and patients’ outcomes.

Patients	Dose CD3+ Infused (×10^7^/kg)			Outcome Post-DLI			Time of Progression	Status at 2 Years Post Start LEN Cycle1
	1	2	3	Acute GVHD	Chronic GVHD	Best Response	Months Post Start LEN Cycle1	
P01	0.5	1	5	0	0	PD	10.7	Alive
P02 *	0.5	1	3.8	0	0	CR	14.0	Alive
P03	0.5	1	5	0	0	CR	no PD	Alive
P04 *	0.5	1	5	0	0	PD	8.8	Alive
P05 *	0.5	1	Relapse	0	0	PD	9.3	Alive
P06 *	0.5	1	5	0	0	PD	9.5	Alive
P07 *	0.5	1	5	0	Moderate (mouth (1), skin (2), liver (2), eyes (1)	PD	9.7	Alive
P08 *	0.5	Relapse	Relapse	0	0	PD	6.7	Alive
P09	0.5	1	Relapse	0	0	PD	9.2	Alive
P10	0.5	0.9	5	0	0	VGPR	18.9	Alive
P11 *	0.5	1	5	0	0	VGPR	20.6	Alive
P12	0.5	1	2.2	I (skin) **	0	VGPR	61.6	Alive
P13	0.5	1	Liver GVHD	0	Mild (oral and liver)	VGPR	no PD	Alive
P14	0.5	1	3	0	Mild (oral and liver)	VGPR	no PD	Alive
P15 *	0.5	Relapse	Relapse	0	0	PD	7.4	Alive
P16	0.9	4.9	4.9	0	0	VGPR	no PD	Alive
P17	0.5	1	7.0	0	0	VGPR	32.9	Alive
P18	0.5	1	5	0	0	VGPR	15.5	Alive
P19 *	0.5	1	5	0	Mild (oral)	VGPR	12.3	Alive
P20	0.5	1	5	0	0	VGPR	no PD	Alive
P21	0.5	1	3.9	0	0	VGPR	40.8	Alive
P22 *	0.5	1	Relapse	0	0	PD	8.3	Dead

CR: complete remission; PD: progressive disease; VGPR: very good partial response. * High-risk cytogenetics. ** During second cycle of lenalidomide and dexamethasone.

**Table 4:** Non-hematologic grade III–V adverse events. Adverse events were defined by Common Terminology Criteria for Adverse Events (version 4.0) and reported between time of first cycle of lenalidomide and dexamethasone to one year after the last DLI or time of progression, whichever came first.

Metabolic	N (%)	Grade
Hypophosphoremia	2 (9)	3
De novo diabetes	1 (4)	3
Gastro-intestinal		
Nausea	1 (4)	3
Anorexia	1 (4)	3
Liver transaminitis	2 (9)	3
Gastro-enteritis	1 (4)	3
Infectious		
Cellulitis	1 (4)	3
Influenza B + Haemophilus influenzae	1 (4)	3
Pneumonia	4 (18)	3
Bronchitis (Serratia)	1 (4)	3
Parainfluenza	1 (4)	3
Respiratory syncitial virus	1 (4)	3
Others		
Basal cell carcinoma	2 (9)	3
Pulmonary embolism	1 (4)	3

**Table 5:** Minimal residual disease kinetics. Values represent absolute numbers and percentage of clonal plasmocytes.

Patient	Pre LEN	Pre DLI#1 (M + 6)	Pre DLI#2 (M + 8)	Pre DLI#3 (M + 10)	M + 15	M + 18	M + 21	M + 36	M + 60	Month of PD	Before New Tx
P01	1144 (0.011%)	Neg	Neg	Neg *						10.7	277 (0.0028%)
P02	20,330,000 (1.9%)	Neg	Neg	11,760 (0.12%)	2231 * (0.023%)					14	4800 (0.049%)
P03	100 (0.001%)	Neg	Neg	Neg	Neg	Neg	Neg	Neg	/	/	/
P04	675,000 (25%)	111,860 (1.19%)	252,200 (2.6%)	507,400 * (5.9%)						8.8	464,100 (5.1%)
P05	6144 (0.064%)	Neg	149 (0.0015%)	ND *						9.3	2,501,000 (61%)
P06	60 (0.0006%)	Neg	Neg	128 * (0.0012%)						9.5	370 (0.0037%)
P07	14,100 (0.15%)	68,640 (0.66%)	218,400 (2.1%)	48,500 * (0.5%)						9.7	/
P08	51,000 (0.51%)	100,800 * (0.96%)	114,240 (1.12%)							6.7	/
P09	Neg	52 (0.0005%)	83 (0.0008%)	2190 * (0.03%)						9.2	/

* Time of progression disease assessment. LEN: Lenalidomide; M: month; ND: not done; Neg: negative; DLI: donor lymphocyte infusion; PD: progressive disease; Tx: treatment.

**Table 6:** Subsequent lines of treatments following Len/Dex/DLIs protocol.

Patients #	Lines of Treatment				
	1	2	3	4	5
P01	DRd				
P02	DRd	KCd	PCd	DCEP	
P03	Remission				
P04	DRd				
P05	KCd	DPd			
P06	DRd				
P07	DRd				
P08	DVd				
P09	DRd	KCd	PCd	Teclistamab	
P10	DRd				
P11	DRd				
P12	DRd				
P13	Remission				
P14	Remission				
P15	DRd	KCd	PCd	D-PACE	Selinexor
P16	Remission				
P17	Len/Dex	DVd	KCd	Cyclo/Dex	
P18	Len/Dex				
P19	KCd	DRd	PCd	DPd	Teclistamab
P20	Remission				
P21	DRd				
P22	Kd	PCd	ICd	Bendamustine	

Cyclo: cyclophosphamide; DCEP: dexamethasone, cyclophosphamide, etoposide, cisplatin; Dex: dexamethasone; D-PACE: dexamethasone, cisplatin, adriamycin, cyclophosphamide, etoposide; DRd: daratumumab, lenalidomide, dexamethasone; DVd: daratumumab, bortezomib, dexamethasone; ICd: ixazomib, cyclophosphamide, dexamethasone; KCd: carfilzomib, cyclophosphamide, dexamethasone; Kd: carfilzomib, dexamethasone; Len/Dex: lenalidomide, dexamethasone; PCd: pomalidomide, cyclophosphamide, dexamethasone; DPd: daratumumab, pomalidomide, dexamethasone.

## Data Availability

The datasets for the current study are not publicly available due to privacy regulations in the Province of Québec. Further inquiries can be directed to the corresponding author.
